# Surface Quality Evaluation of 3D-Printed Carbon-Fiber-Reinforced PETG Polymer During Turning: Experimental Analysis, ANN Modeling and Optimization

**DOI:** 10.3390/polym16202927

**Published:** 2024-10-18

**Authors:** Anastasios Tzotzis, Dumitru Nedelcu, Simona-Nicoleta Mazurchevici, Panagiotis Kyratsis

**Affiliations:** 1 Department of Product and Systems Design Engineering, University of Western Macedonia, 50100 Kila Kozani, Greece; 2 Department of Manufacturing Engineering, “Gheorghe Asachi” Technical University, 700050 Iasi, Romania; dnedelcu@tcm.tuiasi.ro (D.N.); simona-nicoleta.mazurchevici@academic.tuiasi.ro (S.-N.M.)

**Keywords:** 3D printing, artificial neural network, carbon fiber reinforced polymer, genetic algorithm, machining, polyethylene terephthalate glycol, surface roughness

## Abstract

This work presents an experimental analysis related to 3D-printed carbon-fiber-reinforced-polymer (CFRP) machining. A polyethylene-terephthalate-glycol (PETG)-based composite, reinforced with 20% carbon fibers, was selected as the test material. The aim of the study was to evaluate the influence of cutting conditions used in light operations on the generated surface quality of the 3D-printed specimens. For this purpose, nine specimens were fabricated and machined under a wide range of cutting parameters, including cutting speed, feed, and depth of cut. The generated surface roughness was measured with a mechanical gauge and the acquired data were used to develop a shallow artificial neural network (ANN) for prediction purposes, showing that a 3-6-1 structure is the best solution. Following this, a genetic algorithm (GA) was utilized to minimize the response, revealing that the optimal combination is 205 m/min speed, 0.0578 mm/rev feed, and 0.523 mm depth of cut, contributing to the fabrication of low friction parts and shafts with a high quality surface, as well as to the reduction of resource waste. A validation study supported the accuracy of the developed model, by exhibiting errors below 10%. Finally, a set of enhanced images were taken to assess the machined surfaces. It was found that 1.50 mm depth of cut is responsible for the generation of defects across the circumference of the specimens. Especially, combined with 150 m/min cutting speed and 0.11 mm/rev feed, more flaws are produced.

## 1. Introduction

Surface roughness is a critical aspect in machining, significantly influencing the performance, functionality, and longevity of machined components. During machining processes such as turning, drilling, milling, or grinding, the surface quality of a part is determined via the intricate interactions between the cutting tool and the workpiece material. Surface roughness, often quantified via parameters such as average surface roughness, represents the deviations of a surface from its ideal form, including microscopic peaks and valleys left by the cutting tool. Several factors affect surface roughness during machining, including the material properties of the workpiece, tool geometry, cutting speed, feed rate, and depth of cut. Additionally, tool wear, vibration, and the presence of lubricants or coolants play crucial roles in determining the final surface finish. Achieving an optimal surface roughness is essential in industries where the quality of the surface directly impacts the performance, such as in aerospace, automotive, and precision engineering. For example, lower surface roughness can enhance fatigue resistance, improve sealing capabilities, and reduce friction between contacting surfaces. Conversely, inadequate control over surface roughness can lead to premature failure, increased wear, and reduced efficiency of components.

Therefore, the surface quality evaluation of manufactured components is a topic that has been widely studied for a high volume of typical materials, such as aluminium alloys and steels. Perez et al. [1] studied the effects of the cutting speed on the generated cutting forces and surface integrity of Al7050-T7451 during face milling. Similarly, Parida et al. [2] examined the influence of cutting speeds and different tool nose radii on various machining aspects of Al6061, including surface roughness. Swain et al. [3] analyzed the cutting tool vibration effect on the tool wear rate and surface roughness, during high speed machining of AISI1040 steel. Dubey et al. [4] measured the surface roughness of machined AISI306 stainless steel under varying lubrication conditions. Besides aluminium and steel, titanium as well as nickel-based alloys are frequently used materials in aerospace, automotive, and similar industries. Therefore, they have been studied to a certain degree as well. Aslantas et al. [5] investigated the behavior of Ti6Al4V titanium alloy during micro-milling, examining the surface roughness variations under dry and different lubrication conditions. A study by Zahoor et al. [6] highlighted the effects of environmentally conscious machining of Inconel 718 on surface quality and tool wear.

With the continuous improvement of the composite materials, the aforementioned industries have made a considerable effort towards the implementation of such materials in their manufacturing processes. As a result, carbon-fiber-reinforced-polymers (CFRPs), aluminium-based composites, and metal matrix composites in general seem to have steadily replaced traditional materials. Bhushan [7] worked on the Al7075/SiC composites for green manufacturing, investigating the impact of the tool nose radius and other cutting factors on the surface roughness and tool wear. Similarly, Singh et al. [8] studied the surface quality of machined Al6061/SiC/Gr composites. Xiong et al. [9] presented a study on milling in situ TiB2/7050Al composite, focusing on the surface roughness and fatigue properties of the machined material. Especially CFRPs constitute an emerging research area related to composite machining, since their properties enable the fabrication of components with increased strength-to-weight ratios. Moreover, according to Geier and Pereszlai [10], CFRPs are difficult to machine and process, in contrast to metal matrix composites, due to the occurrence of surface issues such as delamination, uncut fibers, small cracks, and protruding fibers. Despite the fact that CFRP machining is a relatively new research topic, the number of available studies for drilling [11,12], milling [13,14,15,16,17], and orthogonal cutting [18] is rapidly growing. This phenomenon is probably due to the increasing demand for models and optimized solutions related to CFRP machining. Besides carbon-fiber-reinforced materials, similar studies have been conducted on composites that benefit from the incorporation of natural or glass fibers. Slamani and Chatelain [19] conducted a review of research on the machinability behavior of fiber-reinforced materials, including both natural and glass fibers. El-Ghaoui et al. [20] focused on the machinability of glass-fiber-reinforced-polymer (GFRP), and especially on the effect of graphene addition on the composite’s matrix, during milling operations. Prasanth et al. [21] investigated the effects of the milling process parameters on the machining force generation and the surface integrity of GFRP parts.

The advent of new manufacturing technologies however, such as additive manufacturing (AM), generated a new topic within the research area of CFRP machinability behavior, that is, the machining of 3D-printed CFRPs, which constitutes an alternative manufacturing method of components. AM, also known as 3D printing, is a revolutionary technology that enables the creation of objects by building them layer by layer, directly from digital models. Unlike traditional subtractive manufacturing, where material is removed from a solid block to form a part, AM adds material precisely where needed, minimizing waste and enabling the production of complex geometries that are difficult or impossible to achieve using conventional methods. This process can use a variety of materials, including polymers, metals, ceramics, and composites, offering flexibility in design and application. AM has gained significant traction in industries such as aerospace, automotive, healthcare, and consumer goods due to its ability to reduce lead times, lower production costs, and facilitate rapid prototyping. Additionally, AM allows for the customization of products, making it ideal for producing patient-specific medical implants or personalized consumer products. As the technology evolves, it is also being integrated into high-volume production [22], advancing the future of manufacturing. However, challenges [23] such as material limitations, surface finish quality, and scalability remain areas of ongoing research and development. Cococcetta et al. [24] investigated the surface finish, tool wear, and burr development during milling of 3D-printed CFRPs. Hassan et al. [25] utilized the finite element method (FEM) to model the CFRP machining. El Mehtedi et al. [26] examined the effects of milling cutting conditions on the surface quality of polyethylene terephthalate glycol (PETG)-based CFRP. Similar studies [27,28,29,30] deal with the post processing of CFRPs, by utilizing equivalent experimentation frameworks, methods, and tools. It is evident that most studies deal with either milling or drilling, by employing mostly pure experimental work and standard analyses, with the exception of a few studies that implemented FEM [25,31,32]. Despite the fact that turning is considered a simpler process compared to milling or drilling, it is still understudied, at least in terms of the 3D-printed CFRPs. Moreover, according to Song et al. [33], turning is one of the most applied post processing operations for cylindrical components made of CFRPs. This paper takes a step further into experimentation of 3D-printed CFRPs, by implementing artificial neural networks (ANNs) and genetic algorithms (GAs) into the modeling procedure. In addition, the study deals specifically with the lathe machining of a PETG-based CFRP, under conditions that fit to post processing operations. PETG is a versatile thermoplastic polymer widely used in both industrial and consumer applications due to its balance of strength, flexibility, and ease of use. As a glycol-modified version of polyethylene terephthalate (PET), PETG is more durable and less brittle, making it ideal for applications requiring toughness and impact resistance [34]. It is also popular in 3D printing due to its ease of extrusion, low shrinkage, and excellent adhesion between layers, resulting in durable and detailed prints. Additionally, it can be thermoformed, molded, and cut easily, allowing for a wide range of manufacturing techniques, making it versatile across industries such as automotive, construction, and electronics industries. PETG is recyclable [35] and more environmentally friendly compared to other plastics, further enhancing its appeal in sustainable production practices.

The utilization of ANN modeling has been widely applied to various engineering areas. Shahgholi et al. [36] utilized neural networks for the prediction of heat transfer in biomedical engineering applications. Moradi et al. [37] developed ANN models for the prediction of mechanical parameters related to 3D-printed ABS specimens. Similarly, Belaadi et al. [38] worked on bio-composite drilling, utilizing ANN modeling. It is evident that several areas of research such as biomedical engineering and 3D printing and machining benefit significantly from the implementation of neural networks in the modeling process, including manufacturing [39,40].

## 2. Materials and Methods

**Figure 1:**
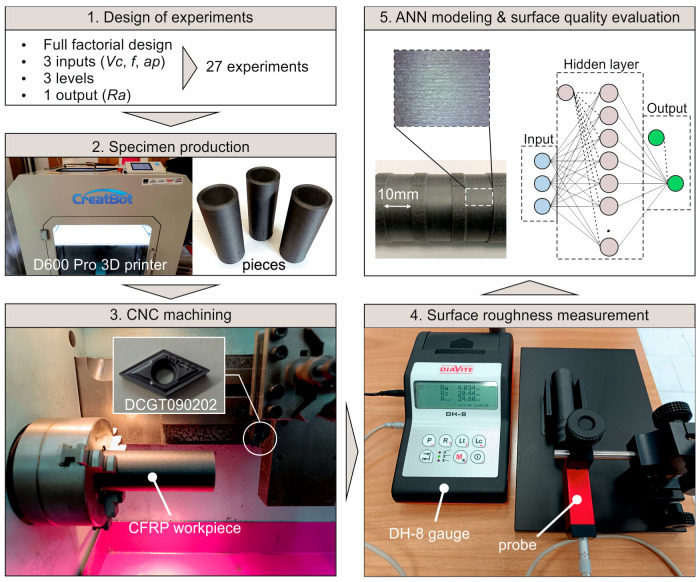
The five basic stages of the study.

The present study deals with a machinability evaluation of a composite filament used in 3D printing, which is based on carbon-fiber-reinforced PETG. As illustrated in Figure 1, the study is divided into five steps. First, the machining experiments were designed according to three critical cutting parameters and their corresponding levels. Next, the cylindrical workpieces were fabricated. During step 3, the machining tests were carried out with the aid of a CNC lathe and appropriate tooling. Next, the surface roughness of the machined pieces was measured according to the corresponding standards, by utilizing mechanical measurement methods. Finally, the collected data were further analyzed by examining enhanced images of the machined surfaces. In addition, the response was modeled with machine learning techniques. The next sections discuss both the experimental framework and the modeling procedure.

### 2.1. Experimental Framework and Apparatus

In this study, CARBON:PLUS filament (Neema3D, Petroupoli, Greece) was used to fabricate nine cylindrical workpieces of 30 mm diameter and 80 mm length each, utilizing a CreatBot D600 Pro 3D printer (Henan Creatbot Technology Limited, Zhengzhou City, Henan Province, China). The filament is a PETG-based material, reinforced with 20% carbon fiber. Table 1 presents the standard properties of the material, which is typically used for automotive parts, drones, and other radio controlled vehicles, since it exhibits increased stiffness, as well as increased impact and heat resistance.

The fabrication parameters are shown in Table 2. Each specimen was fabricated in such a way so that the outer walls were solid and of suitable thickness, allowing the cutting tool to machine over a solid and continuous surface, avoiding any gaps that might be present between the bonding of each wall layer. According to Patel et al. [42], the combination of 0.2 mm layer thickness and 40 mm/s speed contributes towards the material bonding and alignment. Therefore, the layer, speed, and filament flow were set with respect to the findings of the previously mentioned study to ensure increased material bonding. The basic parameters, such as nozzle and build plate temperatures, were set in accordance to the manufacturer’s recommendations. Finally, the infill density and pattern were set to 50% and rectilinear, respectively, to ensure a firm grip of the workpiece on the chuck.

To machine the specimens, a Boxford 160TCL CNC lathe (Boxford Holdings Ltd., Halifax, West Yorkshire, UK) and a set of 55° diamond-shaped (Sumitomo Electric Hardmetal Corporation, Itami City, Hyogo Prefecture, Japan), positive inserts with a 0.2 mm nose radius were utilized. The dry machining conditions are given in Table 3, which were selected according to the tool manufacturer for light cutting to finishing operations. *Vc* denotes the cutting speed, *f* represents the feed rate, and finally, *ap* is the depth of cut. It should be noted that the specified range of conditions are typically applied to pure polymers [43] such as ABS, PETG, and PLA as well. The designation codes for both the cutting inserts and the tool holder are DCGT090202N-SC and SDJCL1010-03S accordingly. The lead angle given by the tool holder is 93°. The selected cutting conditions and their combinations, according to a full factorial design, led to 27 cutting experiments. Three cuts, 10 mm wide each, were machined on each one of the 9 test pieces, with respect to the cutting condition combinations (Table 4).

Finally, the surface roughness of the machined specimens was measured, in terms of the average surface roughness, *Ra*. The measurements were carried out with a DIAVITE DH-8 gauge system (Diavite AG, Bülach, Switzerland), following the norms indicated in the ISO 21920-2 standard. The final value of *Ra* was computed as the average of four measurements, taken at equivalent anti-diametral points of each machined circumference. Since a small nose radius was used and relatively slow feeds, it was expected that the roughness values would be measured at a typical range for turning. Therefore, with the expected surface roughness magnitude in mind, as well as considering the probe’s tip radius, the cut-off length, λc, was set to 0.8 mm, as suggested by the ISO 21920-3 guidelines.

### 2.2. Experimental Design and Acquired Data

The selected cutting conditions and their levels yielded a set of 27 tests, by employing the full factorial design. The specific experimental design is widely used for the evaluation of several engineering processes including machining [44,45,46,47]. Its benefits are derived from the fact that all combinations of conditions are tested and none are skipped, improving the reliability of the modeling procedure. Table 4 contains the *Ra* values for each one of the 27 experiments, including the corresponding cutting conditions. It was found that the magnitude of the measured data is similar to the findings of Cococcetta et al. [24], for the 3D-printed nylon CFRP slot milling under dry conditions. An indicative range includes values between 2.78 μm and 4.03 μm, depending on the milling technique. Other studies reported similar values of *Ra* for the dry drilling of epoxy laminate CFRP [26], revealing measurements ranging from approximately 3 μm to 5 μm when using twist drills at low feed rates. In addition, for the orthogonal cutting of the epoxy prepreg CFRP [48], the study showed *Ra* values between 0.47 μm and 2.81 μm for light cuts.

### 2.3. Artificial Neural Network Development

ANNs are computational models inspired by the human brain’s network of neurons. These networks consist of interconnected nodes, called neurons, which work together to process and learn from data. They are particularly effective in tasks such as image and speech recognition, as well as predictive analytics, making them ideal to model complex, non-linear relationships in data. By adjusting the connections and weights between neurons, ANNs learn to perform tasks by minimizing errors, often through a process called backpropagation. An increased number of studies that are available in the literature support the efficiency of ANNs when modeling similar processes [38,39,40].

**Figure 2:**
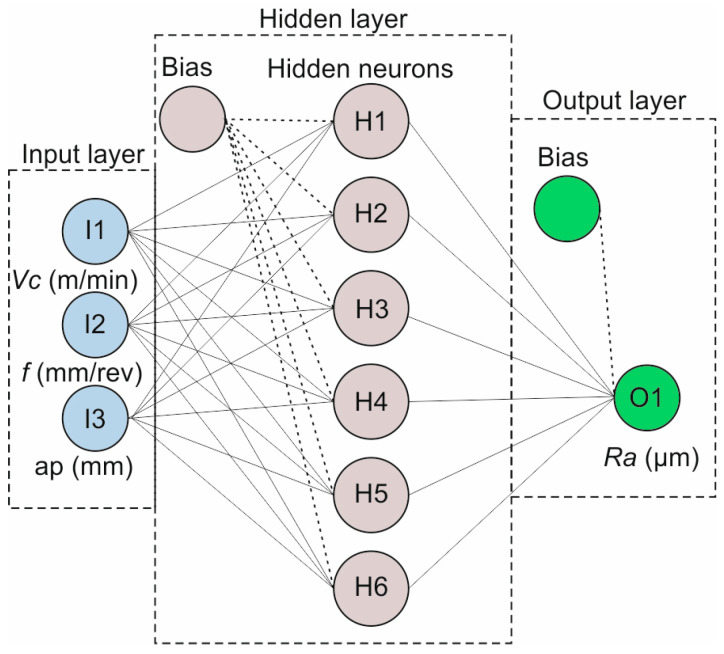
The 3-6-1 ANN structure for the study.

The feedforward, backpropagation method was used in the present study, implemented with the Levenberg and Marquardt (LM) [49] training algorithm. The LM and the Broyden–Fletcher–Goldfarb–Shanno (BFGS) algorithms are the most commonly applied algorithms for the supervised learning of function approximation problems. Especially the LM algorithm is usually the fastest, despite the fact that it requires more memory compared to other algorithms, and is commonly acknowledged as the first-choice supervised algorithm. Therefore, to identify the most suitable algorithm for the surface roughness approximation, a preliminary test was carried out to evaluate their accuracy, as shown in the work by Erkan et al. [50]. Table 5 presents a comparison between the two algorithms, for a wide range of structures. The comparison was made in terms of the generated correlation coefficient *R*-value and the root mean squared error (RMSE), revealing that the 3-6-1 structure with the LM algorithm is the best solution. The *R*-value is a metric that determines the proportion of the variance in the output of the model and ranges between 0 and 1. The higher the *R*-value, the better the model fits the target dataset. Similarly, RMSE is a metric that describes how far apart the predicted points are from the observed points in the dataset. Therefore, the lower the RMSE, the better the data fit. The RMSE was calculated with Equation (1) for *N* number of experiments, with *R_a(exp)_* denoting the experimentally measured surface roughness and *R_a(sim)_* the predicted one from the ANN model. The experimental data used to feed the networks (Table 4) were divided into three groups. The first group, comprising 70% of the experimental data, was used to train the model. The second group, containing 15% of the data, was used to validate the model, and the remaining 15%, belonging to the third group, was used for testing purposes. The learning rate parameters [51] were set to standard values. Therefore, the maximum number of training epochs was set to 1000, the epochs between display to 25, the maximum validation checks to 100, and the minimum performance gradient to 10^−7^. Finally, Figure 2 illustrates the used ANN structure.

(1) $$RMSE=\sqrt{\frac{\sum_{i=1}^{N}{\left(R_{a(\exp)}-R_{a(sim)}\right)}^{2}}{N}}$$

The hyperbolic tangent (tanh) function was selected for the node output calculation, because it is considered more suitable for the training process of the specific modeling procedure [52]. Additionally, the data mapping ranges between −1 and +1. Therefore, the normalization of the data was made with respect to the aforementioned range [−1, 1], using Equation (2), where *y_normalized_* is the normalized value of either the input or output, as a function of the actual inputs (*V_c_*, *f*, *ap*) and measured outputs (*R_a_*). *y_max_* and *y_min_* represent the maximum and minimum actual value of either the inputs or output data. To describe the mathematical expression of the tanh activation function, Equation (3) was used, with tanh being the hyperbolic tangent. The calculation formula for the output variable, based on the inputs and their weights, is given via Equation (4). *R_a_* denotes the output variable, herein the average surface roughness, *W_i_* are the output layer weights for each of the *n* hidden nodes, *f*(*x*) is the tanh activation function for the hidden neuron *H_i_* value, and finally, *b* represents the bias of the output layer.

(2) $$y_{normalized}=\frac{2}{y_{\max}-y_{\min}}\times y-\frac{y_{\max}+y_{\min}}{y_{\max}-y_{\min}}$$

(3) $$f(x)=\tanh(x)=\frac{2}{1-e^{-2x}}-1$$

(4) $$R_{a}=\sum_{i=1}^{n}W_{i}f(H_{i})+b$$

The final formula that can be used for predicting the surface roughness under the range of conditions of Table 3 is Equation (5). This formula was generated via the selected 3-6-1 ANN model, including the hidden neurons and the model bias. Each hidden neuron *H_i_* value can be determined with Equations (6)–(11). The hidden neuron formulas were produced by taking into account both the weights of the input layer and the biases of the hidden layer.

(5) $$R_{a}=0.53949H_{1}+0.59002H_{2}+0.79014H_{3}+0.24072H_{4}-0.56114H_{5}+1.1271H_{6}+1.2983$$

(6) $$H_{1}=\tanh(0.5\times (0.89122V_{c}-1.12383f+1.5976ap-2.7544))$$

(7) $$H_{2}=\tanh(0.5\times (2.5255V_{c}-0.14801f+0.27643ap-1.6012))$$

(8) $$H_{3}=\tanh(0.5\times (-1.8391V_{c}+1.8792f+0.28126ap+0.73498))$$

(9) $$H_{4}=\tanh(0.5\times (0.75443V_{c}+1.8957f-0.52196ap+0.85881))$$

(10) $$H_{5}=\tanh(0.5\times (0.91554V_{c}-2.2153f-0.476251ap+1.5837))$$

(11) $$H_{6}=\tanh(0.5\times (-0.73428V_{c}+1.5407f+1.0832ap-3.4194))$$

**Figure 3:**
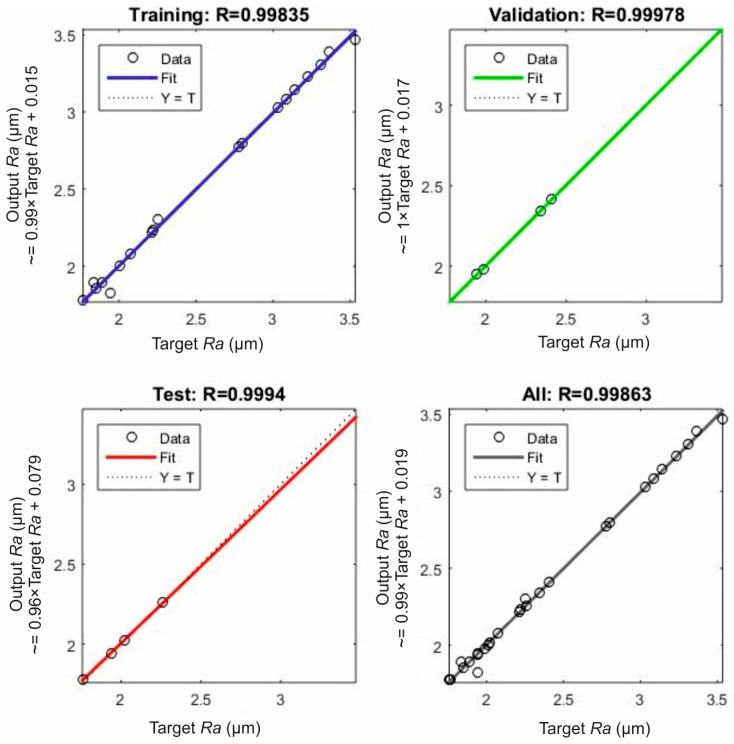
The regression plots for each of the data sets: training set, validation set, test set, and the summation of the data.

Figure 3 illustrates the regression plots for the modeled surface roughness. Each plot corresponds to a data set according to the grouping done during the ANN development. By observing the plots, the strong fit between the experimental and the modeled surface roughness is evident. First of all, it is shown that the majority of the predicted data points are on the zero-error line (Y = T), with deviations being scarce. Second, the fit line and the error line are collinear, proving the increased accuracy. Moreover, the high coefficient *R* values generated further support the high levels of accuracy of the model. This fact contributes towards the computation of low errors. Finalizing, as shown in Figure 3, the coefficient *R* for the training data set, the validation, the testing, and the summation of the data were calculated as equal to 0.99835, 0.99978, 0.99940, and 0.99863, respectively.

## 3. Results and Discussion

In this section, the effect of each parameter on the measured output is analyzed. In addition, the interaction between the parameters is visualized for each level, with the corresponding plots.

### 3.1. Analysis of the Variables’ Effect

**Figure 4:**
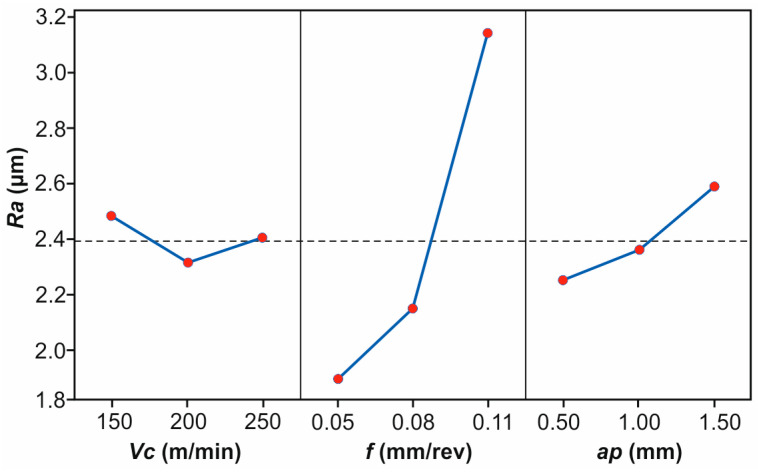
The mean effects plot for the measured surface roughness.

Figure 4 illustrates the mean effects plot, revealing the individual effect of each variable on the *Ra*. It is evident that an increase in *Vc* from 150 m/min to 200 m/min decreases *Ra* noticeably. On the contrary, further increasing *Vc* to 250 m/min increases *Ra*; however, the effect is lighter. Low speeds probably produce insufficient heat to effectively soften the bonding matrix, leading to a defective surface. In a similar manner, higher speeds, especially after a certain point, are responsible for the generation of excessive amounts of heat, which generally tend to deteriorate the surface. The overall improvement in the surface finish is evident from 150 m/min to 200 m/min, although it is not that significant compared to the effect of feed. Similar results were reported in studies related to various materials processing methods such as AISI H11 steel turning [53], aluminium turning [54], and CFRP milling [26]. *Ra* increases significantly when changing *f* from 0.05 mm/rev to 0.08 mm/rev, an effect which is expectable, since higher feeds lead to larger tool marks on the surface, resulting in greater roughness. In addition, increasing the feed to the next level (0.08 mm/rev to 0.11 mm/rev), results in *Ra* continuing to increase sharply, supporting the fact that increased *f* deteriorates the surface finish of a component. Finally, the overall shift from 0.05 mm/rev to 0.11 mm/rev dramatically affects *Ra*. With more than double the feed rate, *Ra* reaches approximately doubled values, compared to 0.05 mm/rev. Especially for the feed effect, similar findings have been reported by a number of studies for CFRP materials [11,15,26], as well as metallic materials [53,55,56], for feeds and speeds of similar magnitude. Regarding *ap*, it is observable that it acts increasingly. A moderate increase of surface roughness is present when increasing the depth from 0.50 mm to 1.00 mm, likely due to the higher forces and potential tool deflection at greater depths. Cutting deeper to 1.50 mm, *Ra* continues to increase, though the change is not as steep as the equivalent change in the feed rate. The overall effect of increasing *ap* from 0.50 mm to 1.50 mm is a moderate increase in surface roughness, reinforcing that shallower cuts help in achieving a finer surface finish. El Mehtedi et al. [26], as well as Abena et al. [48], reported that deeper cuts deteriorate the surface quality of CFRPs.

Summarizing, these observations suggest that *f* is the most critical factor affecting roughness during post processing, followed by *ap*, with *Vc* having the least impact in this particular range, with a varying effect.

**Figure 5:**
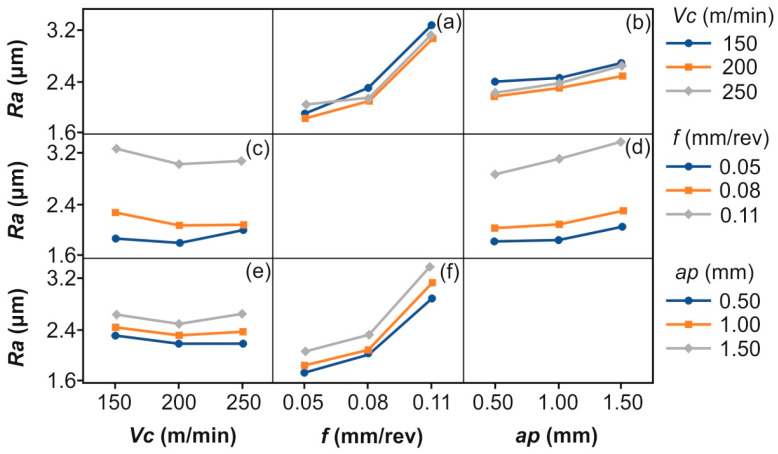
Interaction plot matrix between the three cutting conditions: (**a**) *Vc-f*, (**b**) *Vc-ap*, (**c**) *f-Vc*, (**d**) *f-ap*, (**e**) *ap-Vc*, and (**f**) *ap-f*.

To further assess the interaction between each pair of the cutting parameters, the interaction plot matrix was employed. Figure 5 displays the interaction of two factors and the combined effect on the response. Specifically, Figure 5a shows the interaction between the cutting speed and feed. There is a strong interaction between the cutting speed and feed. At lower feed rates, *Ra* remains fairly consistent across all cutting speeds. However, as the feed increases, particularly from 0.08 mm/rev to 0.11 mm/rev, the difference in *Ra* for different cutting speeds becomes more noticeable, with higher cutting speeds generally resulting in lower roughness. This indicates that the choice of cutting speed becomes more critical at higher feed rates. However, it is noted that 250 m/min cutting speed produces higher *Ra* values at low feed rates. Figure 5b illustrates the interaction between the cutting speed and depth of cut. Depth of cut has a more substantial effect on *Ra* than cutting speed in this interaction. The lines are fairly parallel, which suggests that the increase in Ra with the depth of cut is relatively consistent across all cutting speeds. Hence, the cutting speed has a limited moderating effect on *Ra* when the depth of cut is varied. Figure 5c confirms the previous interaction: the effect of the feed rate on *Ra* is more pronounced at higher feed rates, and higher cutting speeds help mitigate this effect to some extent. This suggests a moderate interaction, where the cutting speed plays a more critical role in controlling roughness when the feed rates are increased. In addition, this interaction revealed that the effect of the cutting speed at 0.05 mm/rev feed is different compared to the effect of the other two feed values. Specifically, it is evident that between 150 m/min and 200 m/min *Ra* decreases; however, when shifting from 200 m/min to 250 m/min, *Ra* rises noticeably. Continuing to Figure 5d, there is a strong interaction between the feed rate and depth of cut. At higher feed rates and greater depths of cut, the *Ra* increases dramatically. The effect of the depth of cut becomes especially important at higher feed rates, making this combination detrimental to the surface finish. Interaction between the depth of cut and cutting speed, shown in Figure 5e, shows that the effect of the depth of cut on *Ra* is relatively consistent across cutting speeds. This suggests that while the depth of cut does influence *Ra*, the cutting speed has a limited impact when combined with the depth of cut changes. Finally, Figure 5f confirms that the combination of high feed rates and large depths of cut leads to much rougher surfaces, meaning that the interaction between these two variables is quite strong.

Summarizing, controlling the feed rate and depth of cut is crucial for minimizing *Ra*, especially at higher levels of these factors. The cutting speed, while important, plays a more secondary role, particularly when the feed and depth are high.

**Figure 6:**
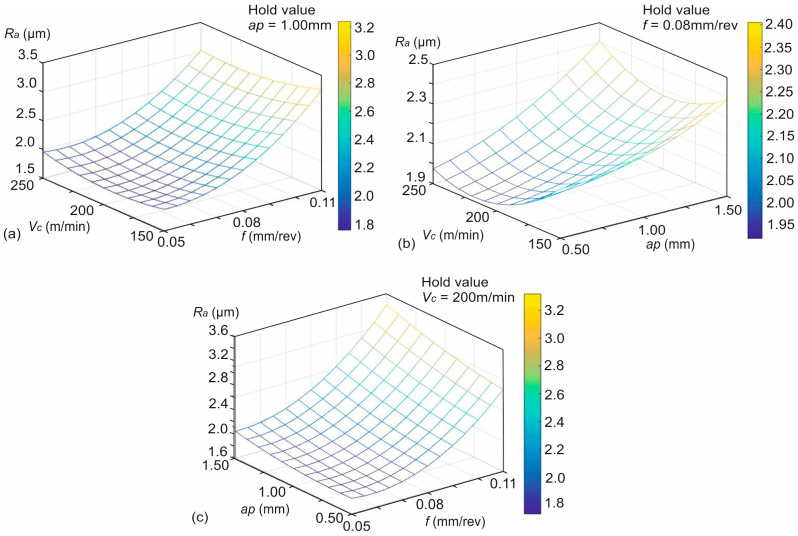
The 3D surface plots for the combined effects: (**a**) *Vc-f*, (**b**) *Vc-ap*, and (**c**) *f-ap*.

To visualize the combined effect of the applied machining parameters, 3D surface plots of Figure 6 were used. These plots illustrate the solutions of the developed Equation (5), with respect to the range of the applied cutting conditions and a defined step, as well as the numerical values of the computed hidden neurons. Figure 6a depicts the solutions for the combination of the cutting speed and feed when holding the depth of cut. Similarly, Figure 6b captures the effects of the cutting speed with the depth of cut on the *Ra*, as well as visualizes the response calculations for the aforementioned combination, by holding the feed. Finally, Figure 6c does the same for the depth of cut and feed, by holding the cutting speed. By observing the plots, it is evident that the combination of high feed rates and increased depths of cuts affects the most the generated *Ra*, further supporting the previously mentioned findings.

In specific, Figure 6a shows that the cutting speed and feed rate exhibit a significant combined influence on roughness. Lowering *f* and increasing *Vc* will result in smoother surfaces. However, at higher feeds, even increasing speed does not fully compensate for the increased roughness. Moreover, an optimal point is revealed close to 200 m/min. Figure 6b highlights that the combination of lower cutting speeds and larger depths of cut is responsible for the generation of higher roughness values. In addition, it is shown that increasing the cutting speed reduces roughness, even at larger depths of cut. However, there is a certain point at around 225 m/min, after which the roughness worsens at *ap* > 1.25 mm. Lastly, the steepest rise in *Ra* is pointed out by Figure 6c, in the region where both the feed rate and depth of cut are high, specifically, at 0.08 mm/rev to 0.11 mm/rev feed and between 1.00 mm to 1.50 mm depth. Moreover, it is shown that the feed rate has the strongest influence on *Ra* in this combination. Even at a constant cutting speed, high feed rates combined with high depths of cut lead to the roughest surfaces.

### 3.2. Function Minimization with the GA

GA optimization is a nature-inspired technique used to solve complex optimization problems, including function minimization. Based on the principles of natural selection and evolution, GAs use a population of candidate solutions that evolve over successive generations. Each candidate, often called a “chromosome”, represents a potential solution encoded as a sequence of genes. The fitness of each chromosome is evaluated via an objective function. In the case of minimization, the goal is to find a solution that reduces the function’s value to its lowest possible point. The genetic algorithm mimics biological processes such as selection, crossover (recombination), and mutation to create new generations of solutions. The fittest solutions, those that yield the lowest function values, are more likely to be selected to produce offspring. Crossover allows the mixing of genetic material between pairs of chromosomes, potentially introducing better solutions. Mutation introduces slight random changes, ensuring diversity in the population and avoiding premature convergence. GAs are particularly effective for nonlinear, non-convex, or high-dimensional problems [57], such as the one presented herein.

To set the algorithm, the parameters shown in Table 6 were used, according to similar studies [58]. The table includes the population size, the mutation and crossover ratios, and the stopping criteria, as well as the variable upper and lower bounds.

**Figure 7:**
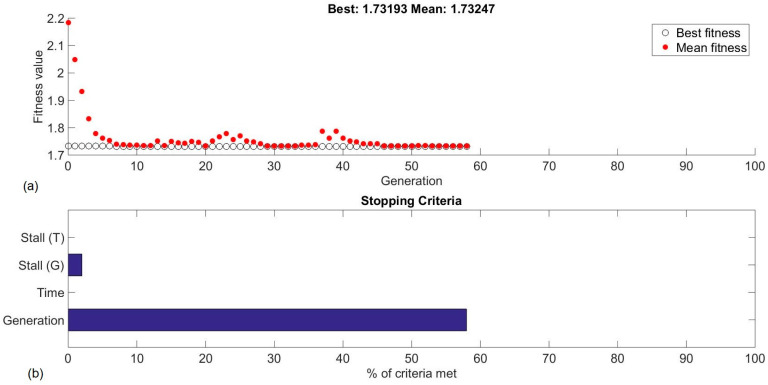
Optimizing response with the GA: (**a**) the fitness value vs generations plot and (**b**) the stopping criteria chart.

Figure 7a illustrates the optimization process, revealing that the optimal surface roughness that can be achieved for the specific PETG-based CFRP during turning is 1.732 μm, at 205.033 m/min cutting speed, 0.0578 mm/rev feed, and 0.523 mm depth of cut. In addition, the generations required to find the solution is 58. Figure 7b depicts the percentage of the stopping criteria that were fulfilled. Since 100 generations were set as the maximum before stopping the algorithm, 58% of the generation criterion was fulfilled. Additionally, the stall generations stopping criterion was set to 50, and the percentage that was covered at the end of the process was 3%. Thus, the stall generations that passed were 6. This stopping criterion detects whether the optimization process is not making meaningful progress toward finding a better solution for a consecutive number of generations (stall generations).

### 3.3. ANN Model Performance and Validation

**Figure 8:**
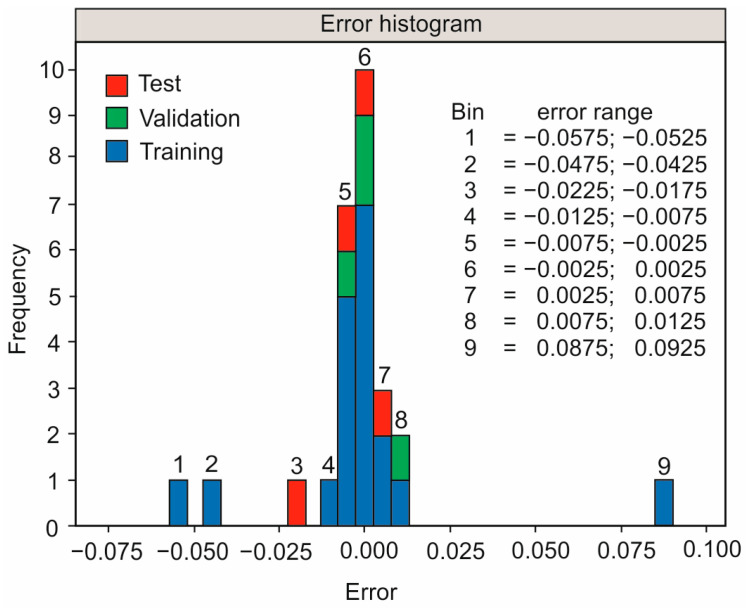
The error histogram for the test, validation, and training data sets.

To analyze the performance of the model, the error ratios were computed. The errors were determined as the difference between the predicted values and the experimental data. Figure 8 illustrates the errors in bins for all three data sets. It is evident that the majority of the errors are around error bin number 6, which crosses the zero-error line. Despite the presence of a few errors farther from this bin, the small errors prove the success of the ANN model training process, as well as the accuracy of the prediction capabilities. It is noted that the summation of the errors is 27, of which 4 correspond to the test step, 4 to the validation step, and 19 to the training process.

To validate the model, three extra experiments were performed and the equivalent measurements were taken. In particular, the optimal solution, which yielded the lowest possible surface roughness, was tested, in addition to two other tests with randomly selected parameters. Table 7 visualizes the comparison between the acquired experimental data and the simulated values for the surface roughness, in terms of the computed relative error. It is observed that the relative error is lower than 10%, supporting the accuracy of the model. It is noted that the error histogram depicts the error difference between the measured and the predicted output values, focusing on the model’s goodness of fit, whereas the relative error of Table 7 is a measure of evaluation of the robustness of the model when predicting output data, generated by randomly selected input variables, which would be the case during a typical machining application.

### 3.4. Machined Surface Assessement

**Figure 9:**
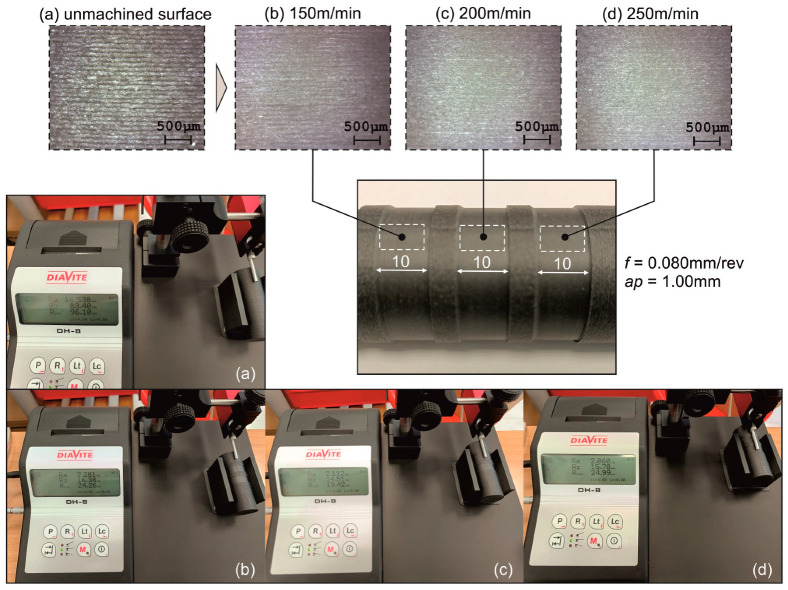
Enhanced images of the unmachined surface and three consecutive cuts, as well as the corresponding measurements, at different cutting speeds: (**a**) unmachined, (**b**) 150 m/min, (**c**) 200 m/min, and (**d**) 250 m/min.

The machined surface of all specimens was evaluated in terms of defects that are related to the material’s nature. First, an indicative evaluation of the machined surface with respect to the three levels of cutting speed was carried out, to visualize the difference in quality between the raw and the machined surfaces of the material. For this reason, a new specimen was machined in the same manner as the rest of the specimens, with the exception that the depth of cut and feed rate remained constant, whereas each cut was machined at a different level of *Vc* (i.e., 150 m/min, 200 m/min and 250 m/min). Figure 9 illustrates the comparison between the unmachined, raw surface of the specimen and the surfaces generated from the varying cutting speed cuts. The cuts were performed at *f* = 0.08 mm/rev and *ap* = 1.0 mm.

By observing the enhanced images, the difference in surface quality is clear when comparing the raw surface (Figure 9a) with the machined ones. As the cutting speed rises, so does the quality of the surface. This difference is more evident between the 150 m/min value (Figure 9b) and either 200 m/min (Figure 9c) or 250 m/min (Figure 9d). In contrast, it is not that apparent between the second and the third level of cutting speed, since no distinct discrepancies are visible. The generalized observations suggest that increased levels of cutting speed tend to decrease the surface roughness. However, the influence is marginal, and thus, it is safe to assume that the suitable range for post processing PETG-based CFRP is between 200 m/min and 250 m/min. In addition, the optimization study described in Section 3.3 revealed that the optimized speed value can be set at approximately 205 m/min.

**Figure 10:**
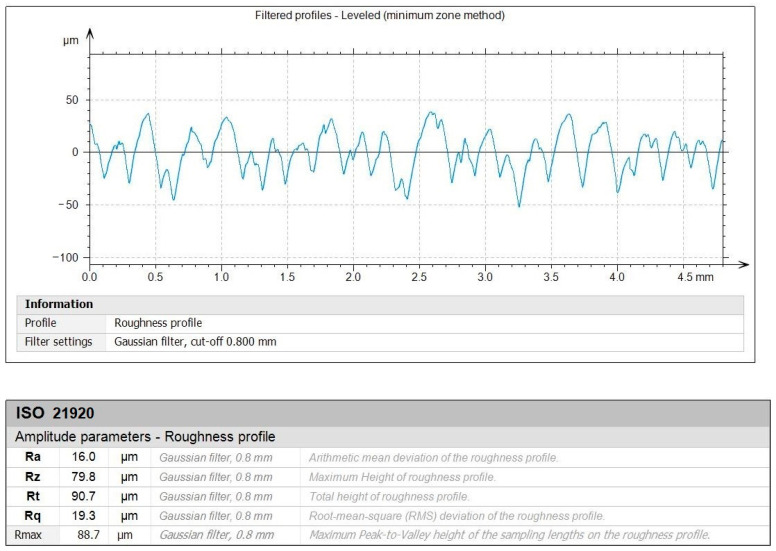
The filtered roughness profile for unmachined surface.

**Figure 11:**
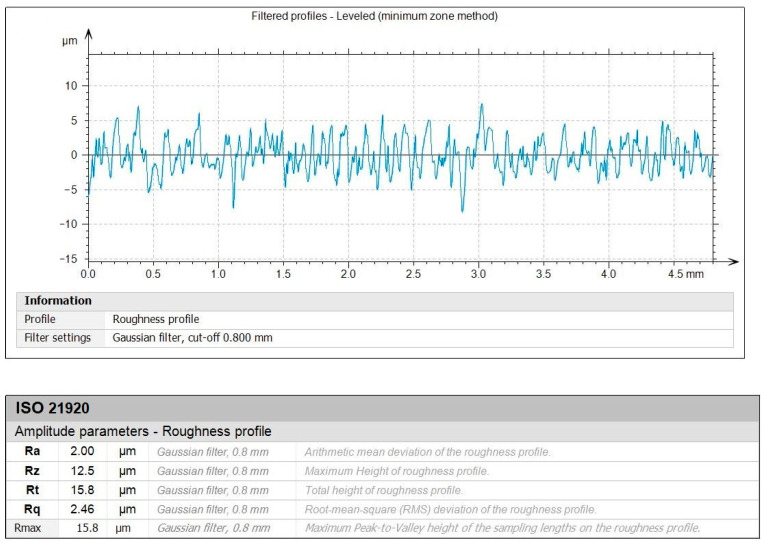
The filtered roughness profile for the *V_c_* = 200 m/min, *f* = 0.08 mm/rev, and *ap* = 1.00 mm combination of cutting conditions.

Figure 10 and Figure 11 depict the roughness profiles for the unmachined surface and the machined surface of an indicative test, respectively, which refers to the previously mentioned test (Figure 9c), with the following settings: *V_c_* = 200 m/min, *f* = 0.08 mm/rev, and *ap* = 1.00 mm. By observing the roughness profiles and their corresponding measurements, a comparison can be made between the material’s raw surface and the generated surface quality after the post processing. It is noted that the average surface roughness, *R_a_*, drops from 16 μm to approximately 2 μm, providing a remarkable improvement to the surface quality. This magnitude of improvement is evident for the other roughness parameters, such as the average maximum height, *R_z_*, and the total height, *R_t_*. Finally, the maximum distance from peak to valley, *R_max_*, is particularly interesting, since it is connected to surface defects such as scratches. Moreover, the fabrication method of the specimens and the nature of the material make the specimens more prone to increased levels of *R_max_*. For the unmachined surface, it was measured to approximately 88.7 μm, whereas for the machined surface it was measured to about 15.8 μm, highlighting the considerable improvement of the surface quality.

Carbon fibers significantly impact the surface roughness of machined CFRP parts due to their unique structure and properties. The high strength and abrasiveness of the carbon fibers may lead to uneven cutting, causing fiber pullout, delamination, and tool wear, all of which contribute to increased values of *Ra* [10]. When machining CFRP, the fibers may not always be cleanly cut but instead be fractured or torn. For this reason, cutting tools with a sharp nose radius are preferred. The polymer matrix, being softer, can be smeared, further complicating the surface finish. Therefore, not all polymers can be used to serve as matrix material for CFRP composites. The PETG in particular, due to its mechanical properties [34], can become an ideal matrix polymer, enabling efficient post processing of parts fabricated with this type of CFRP.

**Figure 12:**
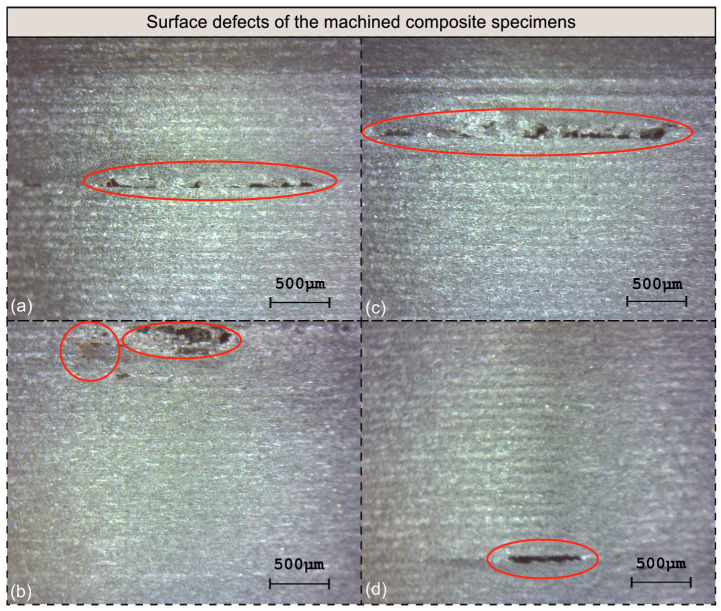
The identified surface flaws on specimens: (**a**) No 2, (**b**) No 6, (**c**) No 9, and (**d**) No 27.

Next, each of the specimens were examined for defects regarding the surface integrity. Figure 12 illustrates the observed flaws. In detail, Figure 12a–d depict the machined surfaces of test numbers 2, 6, 9, and 27, respectively. It is shown that all surfaces share a common flaw, that is, the material being scraped. Surface number 2 (Figure 12a) exhibits a propagation of material scraping of approximately 1.8 mm long, whereas surface number 6 (Figure 12b) suffers from material scraping of an area 750 μm long, with a maximum width of 215 μm, which is accompanied by a discolored area nearby. Surface number 9 (Figure 12c) exhibits a defect of similar pattern to the one observed on surface number 2, with a length of approximately 2.3 mm. Finally, an almost 500 μm long scratch is evident on surface number 9 (Figure 12d). Three out of four surfaces were machined at a depth of cut equal to 1.50 mm, suggesting that deeper cuts probably lead to the generation of scratches along the circumference of the workpiece as a result of higher cutting forces, tool deflection, and increased vibrations. In addition, with the exception of one test, the other three surfaces were machined at 150 m/min speed. Abena et al. [48] highlighted the generation of significantly higher cutting forces, at a magnitude of 100 N to 300 N, for depths of cut lower to the ones applied in this study. Recent findings highlighting the effect of increased feed rates on the generated forces have been reported by Ge et al. [59] for similar carbon-fiber-reinforced materials.

In general, most of the identified defects were measured at the millimeter range. The machining parameters that probably amplify the generation of these flaws are a cutting speed equal to 150 m/min, high feed rates, and a depth of cut equal to 1.50 mm.

## 4. Conclusions

The present research deals with the experimentation of a PETG-based CFRP material under light operations carried out with a CNC lathe. Specifically, the overall surface quality of the material, with respect to standard machining conditions, was evaluated and a model with the machine learning method was developed. The *Ra* was measured, and the produced surface flaws were identified, revealing the next conclusions:

- The cutting speed only slightly affects *Ra*, especially compared to the feed rate. However, its effect cannot be considered unnoticeable. It was found that higher speeds contribute towards smoother surfaces, especially at the range between 200 m/min and 250 m/min.
- The low cutting speed level was responsible for *Ra* measurements averaged at around 2.5 μm. In addition, three out of four defected surfaces were machined at 150 m/min speed.
- Regarding feeds, the 0.05 mm/rev feed was identified as the most beneficial for the generated surfaces. Contrarily, higher feed rates affect surface quality negatively. In particular, the highest value of feed was always responsible for the production of high *Ra* levels, most of the time exceeding values equal to 3.0 μm.
- The depth of cut was identified to be a moderately influencing factor. With an increasing depth of cut, *Ra* seems to steadily rise. However, a depth of cut equal to 1.50 mm was the most detrimental to the surface quality. First, it acts increasingly, yielding values of *Ra* averaged at around 2.6 μm. Second, it is responsible for the development of defects related to material scraping around the circumference of the workpiece.
- The combination of 205 m/min cutting speed, 0.0578 mm/rev feed, and 0.523 mm depth of cut was determined to be the optimal in terms of the generated *Ra*. The specific prediction generated an *Ra* value of 1.732 μm and a value for the equivalent experiment of 1.673 μm.
- The worst condition combination, in terms of the surface quality, was identified to be 150 m/min speed, 0.11 mm/rev feed, and 1.50 mm depth of cut, generating *Ra* equal to 3.534 μm.
- The 3-6-1 structure was selected for the developed ANN model and the LM algorithm for the training procedure. Its correlation coefficient and RMSE were computed to be equal to 0.99863 and 0.028295, respectively, whereas the highest absolute value calculated during the validation process was 9.6%.
- In general, the observed flaws were developed during deep cuts (1.50 mm) at 150 m/min cutting speed.

Overall, the findings of this study can be used in the industry as guidelines for the achievement of optimal roughness levels during CNC lathe machining of 3D-printed CFRP parts. Moreover, the observations related to the surface defects can contribute towards material waste reduction and productivity increase.

Future work will include a thorough microscopic investigation of the surface integrity and the identification as well as the classification of the surface defects, which will benefit from the development of deep neural networks. In addition, research on the phenomena responsible for the defect generation will be carried out. Finally, additional future research can be extended to similar composite filaments, including fiberglass-reinforced materials.

## Figures and Tables

**Table 1:** CARBON:PLUS mechanical and thermal properties [41].

Property	Value	Test Method
Relative density	1.19 g/cm^3^	ASTM D792
Elastic modulus (1 mm/min)	3.8 GPa	ISO 527
Yield stress (50 mm/min)	52.5 MPa	ISO 527
Yield strain (50 mm/min)	4.2%	ISO 527
Strain at break (50 mm/min)	8%	ISO 527
Impact Strength (Izod Notched 23 °C)	3.8 kJ/m^2^	ISO 180-1A
Heat deflection temperature	80 °C	ASTM D648

**Table 2:** Specimen fabrication parameters.

Parameter	Value
Nozzle temperature	255 °C
Build plate temperature	70 °C
Filament diameter	1.75 mm
Nozzle diameter	0.6 mm
Layer	0.2 mm
Speed	40 mm/s
Filament flow	100%
Outer wall layers	4
Infill density	50%
Infill pattern	Rectilinear

**Table 3:** Cutting conditions for the turning operation.

Level	*Vc* (m/min)	*f* (mm/rev)	*ap* (mm)
+1	250	0.11	1.5
0	200	0.08	1.0
−1	150	0.05	0.5

**Table 4:** Design of experiments and surface roughness testing results.

Test	*Vc* (m/min)	*f* (mm/rev)	*ap* (mm)	*Ra* (μm)
1	150	0.05	0.50	1.853
2	150	0.05	1.00	1.834
3	150	0.05	1.50	1.986
4	150	0.08	0.50	2.224
5	150	0.08	1.00	2.258
6	150	0.08	1.50	2.409
7	150	0.11	0.50	3.085
8	150	0.11	1.00	3.229
9	150	0.11	1.50	3.534
10	200	0.05	0.50	1.766
11	200	0.05	1.00	1.758
12	200	0.05	1.50	1.941
13	200	0.08	0.50	2.009
14	200	0.08	1.00	2.076
15	200	0.08	1.50	2.211
16	200	0.11	0.50	2.797
17	200	0.11	1.00	3.034
18	200	0.11	1.50	3.312
19	250	0.05	0.50	1.891
20	250	0.05	1.00	1.943
21	250	0.05	1.50	2.254
22	250	0.08	0.50	1.944
23	250	0.08	1.00	2.022
24	250	0.08	1.50	2.342
25	250	0.11	0.50	2.781
26	250	0.11	1.00	3.144
27	250	0.11	1.50	3.367

**Table 5:** Structure trials for the ANN model, with respect to two training algorithms (bold indicates the best values).

	*R*-Value		*RMSE*	
Structure	LM	BFGS	LM	BFGS
3-3-1	0.98728	0.99576	0.087714	0.050846
3-4-1	0.99604	0.99652	0.050925	0.047260
3-5-1	0.99689	0.99366	0.044322	0.063737
3-6-1	**0.99863**	0.98849	**0.028295**	0.087433
3-7-1	0.99450	0.98157	0.059110	0.118728
3-8-1	0.96962	0.98774	0.140611	0.085909
3-9-1	0.99403	0.99848	0.060037	0.030524

**Table 6:** GA setting parameters.

Parameter	Value
Population size	20
Mutation ratio	0.8
Crossover ratio	0.2
Maximum generations	100
Stall generations	50
Number of variables	3
Lower bound	[150; 0.05; 0.50]
Upper bound	[250; 0.11; 1.50]

**Table 7:** Validation experiments and comparison with simulated values.

Test No	*Vc* (m/min)	*f* (mm/rev)	*ap* (mm)	*R_a, exp_* (μm)	*R_a, sim_* (μm)	Relative Error (%)
1	205	0.058	0.52	1.673	1.732	−3.5
2	225	0.10	0.50	2.548	2.303	9.6
3	175	0.08	0.80	2.035	2.186	−7.4

## Data Availability

Data are contained within the article.
